# Impact of Pharmacist-Conducted Comprehensive Medication Reviews for Older Adult Patients to Reduce Medication Related Problems

**DOI:** 10.3390/pharmacy6010002

**Published:** 2017-12-31

**Authors:** Whitney J. Kiel, Shaun W. Phillips

**Affiliations:** 1 Bronson Methodist Hospital, 601 John St. Suite M-020, Kalamazoo, MI 49007, USA; 2 Clinical and Pharmacy Services, Bronson Healthcare Group, 300 North Avenue, Battle Creek, MI 49017, USA; phillish@bronsonhg.org

**Keywords:** pharmacist, comprehensive medication review, polypharmacy, medication-related problems, medication therapy management, geriatric, START/STOPP criteria, interdisciplinary team

## Abstract

Older adults are demanding increased healthcare attention with regards to prescription use due in large part to highly complex medication regimens. As patients age, medications often have a more pronounced effect on older adults, negatively impacting patient safety and increasing healthcare costs. Comprehensive medication reviews (CMRs) optimize medications for elderly patients and help to avoid inappropriate medication use. Previous literature has shown that such CMRs can successfully identify and reduce the number of medication-related problems and improve acute healthcare utilization. The purpose of this pharmacy resident research study is to examine the impact of pharmacist-conducted geriatric medication reviews to reduce medication-related problems within a leading community health system in southwest Michigan. Furthermore, the study examines type of pharmacist interventions made during medication reviews, acute healthcare utilization, and physician assessment of the pharmacist’s value. The study was conducted as a retrospective post-hoc analysis on ambulatory patients who received a CMR by a pharmacist at a primary care practice. Inclusion criteria included patients over 65 years of age with concurrent use of at least five medications who were a recent recipient of a CMR. Exclusion criteria included patients with renal failure, or those with multiple providers involved in primary care. The primary outcome was the difference in number of medication-related problems, as defined by the START and STOPP Criteria (Screening Tool to Alert doctors to Right Treatment/Screening Tool of Older Persons’ Prescriptions). Secondary outcomes included hospitalizations, emergency department visits, number and type of pharmacist interventions, acceptance rate of pharmacist recommendations, and assessment of the pharmacist’s value by clinic providers. There were a total of 26 patients that received a comprehensive medication review from the pharmacist and were compared to a control group, patients that did not receive a CMR. The average patient age for both groups was 76 years old. A total of 11 medication-related problems in the intervention group patients were identified compared with 24 medication-related problems in the control group (*p*-value 0.002). Pharmacist-led comprehensive medication reviews were associated with a statistically significant different in the number of medication-related problems as defined by the START and STOPP criteria.

## 1. Introduction

Older adults demand increased healthcare attention with regards to prescription medication use due to highly complex regimens and increased vulnerability to poor health outcomes [1,2]. With the advancing age of the Baby Boomer generation, who are now hitting 65 years and older, elderly patient prescriptions now account for over 33% of all prescription medications [3]. As the average life span continues to rise, greater numbers of prescriptions are needed to manage the chronic disease states and conditions commonly encountered in the elderly patient population. Concerns arise due to the fact that medications often have a more pronounced effect on older adults. These effects may include exacerbated confusion, an increased risk of falls, and other adverse drug reactions, further impacting healthcare utilization and increasing costs. The Centers for Medicare and Medicaid Services have provided for medication reviews as part of the Medicare Modernization Act of 2003. These comprehensive medication reviews (CMRs) fall under medication therapy management (MTM) services able to be conducted and billed for by a pharmacist [4]. 

CMRs optimize medications for elderly patients and avoid inappropriate medication use. Using a pharmacist to conduct these reviews saves the physician time and utilizes a medication expert to identify potential problems with a patient’s medication regimen. With the push for healthcare reform, many incentives are being introduced to provide comprehensive health services that improve the quality of health care for patients in non-acute settings. Many have chosen to adopt an interdisciplinary team approach to conquering the advancement in health care services. The American Society of Health-System Pharmacists introduced their Pharmacy Practice Model Initiative in order to guide others in placing pharmacists within these clinical interdisciplinary teams [5]. Previous literature has examined the impact of a pharmacist, conducting such geriatric medication reviews with the goal of improving comprehensive health services for the elderly [2,5].

Various tools have been used to estimate the appropriateness of medications. Arguably the most widely recognized set of criteria is the Beers Criteria for potentially inappropriate medication use in older adults. In 2012, and subsequently in 2015, the American Geriatric Society released the much-anticipated updates to the Beers Criteria [6,7]. While both updates made significant improvements—including updated renal dosing recommendations and a higher level of clinical evidence—other criteria have been proposed to aid in identifying medication related problems. One such criteria is the “Screening Tool to Alert doctors to the Right Treatment and the Screening Tool of Older Persons’ potentially inappropriate Prescriptions” (START/STOPP) Criteria. This tool goes a step further from the Beers list in addressing medications possibly indicated for older adults, as well as expanding on those that should be taken away [8,9]. 

The START/STOPP criteria were used to comprehensively evaluate the appropriateness of medication regimens in older adults in a study by Brahmbhatt et al. [8]. While this study found a significant reduction in the STOPP score between initial and follow up medication reviews, it did not find a significant increase in the START score. The authors commented on the difficulty in initiating medications in the elderly with already complex medication regimens and noted that not all of the criteria are clearly defined. In addition, the study population included home-based veterans, many of whom receive comprehensive healthcare services through other programs. The overarching goal of ‘using screening’ tools such as the START/STOPP criteria is to help identify potential medications that have an increased risk for falls and adverse effects on the elderly. Through management of their complex, numerous medications, many adverse effects due to multiple medications can be prevented [6,8,9].

Polypharmacy can be defined as taking at least five medications on a regular basis [10]. On average, geriatric patients take eight medications daily, greatly increasing the risk of falls or other adverse events. Falls are a particular concern since they are one of the most common threats to independence for elderly patients. Weber and colleagues found a significant reduction in both total number of medications and number of psychoactive medications in their evaluation of standardized medication reviews in an ambulatory patient population. They further looked at the number of falls among their patients, with the intervention group almost 60% less likely to have one or more fall-related diagnosis. Additional studies have shown reductions in adverse drug reactions, high-risk medications, and falls [3,10,11,12,13].

Many studies have sought to improve the appropriateness of medications or decrease medication-related problems. In a study of medication reviews by Vink et al., pharmacists successfully identified medication related problems (MRPs). They showed that 28% of the 232 total MRPs identified in 380 patients were associated with suboptimal therapy, while another 24% consisted of unnecessary medications. Roth and colleagues showed a significant reduction in mean number of medication related problems per patient in their study of 64 elderly patients who were cared for by a pharmacist within a primary care practice. They also noted a significant reduction in acute healthcare utilization of 35%. In a randomized controlled trial by Hanlon and colleagues evaluating medication appropriateness, the inappropriate prescribing scores were reduced significantly in the pharmacist intervention group (24% vs. 6% *p* = 0.0006). They also found a significant acceptance rate from physicians to the pharmacist recommendations in the intervention group (*p* < 0.001) [2,10,14,15].

As indicated in the reports above, a successful comprehensive medication review program would need to impact a wide range of factors in order to address the efficacy and safety of geriatric patients’ medications. Studies have shown improvements in clinical outcomes such as blood pressure as well as patient-centered outcomes including adherence and knowledge of medication therapy. As health care costs rise, essential economic outcomes including length of stay and readmission rates are necessary to evaluate among geriatric patients, who are at an especially high risk for readmission. While literature concerning these outcomes is more limited, a study of team-based care [16] including a pharmacist showed a significant reduction in 30-day readmission rates in the intervention group (10% vs. 38.1%, *p* = 0.04); however, these results were not sustained at 60 days post-discharge [10,16,17].

The promising results of current literature have demonstrated that pharmacists have a unique opportunity to help provide comprehensive medication assessments for older adults. With the potential advantages of impacting medication appropriateness, adverse effects, and potentially increased risk of falls, the next step is to identify the best method of delivering these services. Current literature is mixed on which patients to target for this service with the intent of providing the best utilization of resources. Concerns over the cost of providing a medication review service may be balanced by demonstrating a positive impact on readmission rates and acute healthcare utilization while improving patient care. Comprehensive medication reviews for older adults qualify as CMS medication therapy management and can further increase reimbursement for a health system. Additional research on how to implement these services in a cost effective manner is necessary to lead others in advancing patient-centered care [4].

Healthcare reform has created a demand for change in the delivery of healthcare services. Encouraging the introduction of the medication expert in providing patient-centered care is an important element in the advancement of delivering quality clinical services. The purpose of this research study was to examine the impact of pharmacist-conducted geriatric medication reviews on reduction of medication related problems within a leading community health system in southwest Michigan [18].

## 2. Methods

### 2.1. Overview of Medication-Review Service

Previously, the hospital worked with the local senior services agency to develop an ambulatory pharmacist geriatric CMR service. This helped to determine a target medical practice with a large volume of geriatric patients. The project was conducted with a licensed pharmacist working in the medical practice approximately one-and-a-half days per week for one month from February to March 2014. 

The pharmacist reviewed the scheduled patients for the day to determine those who would benefit from a CMR. After discussing with the patient’s provider, the pharmacist offered to review medications with the patient. Patient’s had the opportunity to accept or decline the service. If accepted, the pharmacist would meet one-on-one with the patient and/or caregivers and review medications, indications, doses, directions, educate on possible side effects, and answer any questions. The pharmacist completed any missing medication information in the electronic medical record for the patient and identified potential medication-related problems to be addressed with the provider. The patients all received an updated medication list upon visit completion and medication education. Medication education included main counseling points: how to take, major side effects, potential drug interactions, and how to avoid potential medication problems.

### 2.2. Study Design and Participants

This study was a retrospective post-hoc analysis of ambulatory patients who received a comprehensive medication review by a pharmacist at a primary care office in southwest Michigan between February and March 2014. Patient inclusion criteria were patients 65 years of age and older, taking at least five prescribed medications, and have received a comprehensive medication review from the pharmacist (intervention group). Exclusion criteria are those patients with renal failure (defined as a creatinine clearance less than 30 mL/min), those with multiple primary care providers, and not meeting the above inclusion criteria. The intervention group patients were compared with a control group from the same medical practice. These patients were identified in the same manner as the intervention group through chart review, but never received an actual review due to limitations further outlined in the discussion section below. This study was approved by the Institutional Review Board of Bronson Health Group. The procedures followed were in accord with the ethical standards of the institution’s committee on human experimentation or with the Declaration of Helsinki, as revised in 2000 (JAMA. 2000; 284:3043–5).

### 2.3. Data Collection

Data was collected through retrospective chart review using Medinformatix, the electronic medical record at the family medicine practice. Information collected for review included demographic information, allergy history, major chronic conditions, complete medication profile, and pharmacist interventions made during the medication review. Information regarding hospitalizations and emergency department visits was collected using Cerner Powerchart for Bronson Battle Creek Hospital. 

### 2.4. Primary and Secondary Outcomes

The primary outcome of the study was the difference in medication-related problems, defined according to the START (Screening Tool to Alert doctors to Right Treatment) and STOPP Criteria (Screening Tool of Older Persons’ Prescriptions). During review, a number was assigned for each criterion the patient met. The number of medication-related problems was identified and totaled after completion of the medication review and compared to the control group from the same time period. 

Secondary outcomes included acceptance of the pharmacists’ recommendations, perceived pharmacist’s value assessed via a Likert-scale survey administered to medical practice providers, and number and type of pharmacist’s interventions. The survey was a paper-based survey with a total of six questions regarding the pharmacist comprehensive medication review service. The survey was distributed and collected by the office administrative staff to each of the 14 primary care providers. Analysis of the survey was completed by the pharmacist, using the mean response to each question. In addition, patients were followed for 90 days after the completion date of comprehensive medication reviews to document hospitalizations and emergency department visits. Pharmacist interventions were categorized according to definition by Cerner PharmNet.

### 2.5. Statistical Analysis

The primary analysis was conducted on all patients having received a comprehensive medication preview and meeting the above inclusion criteria. Assessment of primary and secondary outcomes was performed using descriptive statistics (frequencies, means, and measures of deviation). For the primary outcome of medication-related problems, inferential statistical testing was performed using the chi-square test, with an a priori alpha of 0.05. Categorical secondary endpoints were also analyzed using the chi-square test and continuous variables were analyzed using the non-paired *t*-test. Data was analyzed using the intent-to-treat principle with last data point carried forward. Statistical testing was performed using Microsoft Excel (2010 Microsoft Corporation, Redmond, WA, USA).

## 3. Results

### 3.1. Demographics

There were 26 patients that met study inclusion criteria and received a comprehensive medication review from the pharmacist. The intervention group was compared to a control group of 26 patients for a total of 52 patients included in the study. Demographic and clinical characteristics of the study population are summarized in Table 1. There were no statistically significant differences between the intervention and control groups. 

### 3.2. Primary Outcome

The primary outcome was difference in number of medication-related problems between the intervention group patients that had received a CMR from the pharmacist during the CMR service in February to March 2014 and the control group, patients that did not receive a CMR. The primary outcome is summarized in Table 2. There were a total of 11 medication-related problems defined by the START/STOPP Criteria identified in the intervention group patients, compared with 24 medication-related problems in the control group. This is a statistically significant difference (*p* = 0.002). These medication related problems are further discussed in more detail under secondary outcomes. It is important to note that severity of medication-related problems was not analyzed during our study.

### 3.3. Secondary Outcomes

#### 3.3.1. Pharmacist Interventions

**Figure 1:**
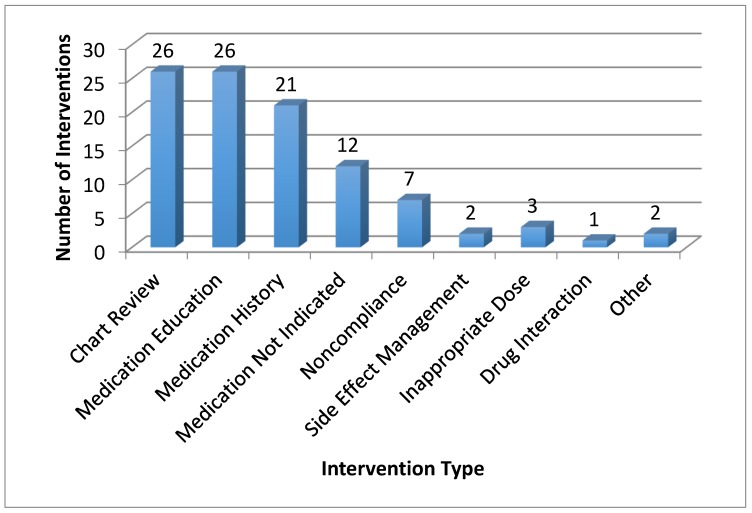
Frequency and Type of Pharmacist Interventions during Pharmacist CMRs.

There were a total of 100 pharmacist interventions made during pharmacist-led comprehensive medication reviews during the study period. Figure 1 displays the number and category for these interventions. The most frequent interventions involved thorough chart review from the pharmacist and medication education for all patients. In addition, the patient’s medication profile was updated for 21 of the 26 patients (81%), which previously had missing or inaccurate information upon completion of their provider appointment. The remaining five patients already had updated medication records upon completion of their primary care provider appointment, which was confirmed through the comprehensive medication review. 

The acceptance rate of pharmacist recommendations for all interventions made was 64% (*N* = 100). The acceptance rate for the primary outcome in regards to recommendations on medication-related problems defined by the START or STOPP Criteria was 35% (*N* = 17). This acceptance rate directly impacts the statistically significant difference identified in our primary outcome. 

#### 3.3.2. Acute Healthcare Utilization

There were a total of seven emergency department visits in the intervention group. Of these seven ED visits, three were fall-related with no resulting admissions for falls. One of the seven ED visits was considered to be medication-related due to noncompliance with blood pressure medications and a resulting admission for uncontrolled hypertension. In the control group, there was a total of six ED visits, one of which may have been medication-related as the patient went to the ED with a chief complaint of difficulty breathing. The patient was recently taken off a diuretic and had a history of congestive heart failure. There were no statistically significant differences in emergency department visits between the two groups. 

A total of three patients were hospitalized during the follow-up period in the intervention group. No admissions could be directly attributed to falls or medications. In the control group, there were four hospitalizations identified during chart review. There were no statistically significant differences in hospitalizations between the two groups. See Table 3 for a summary of acute healthcare utilization.

**Figure 2:**
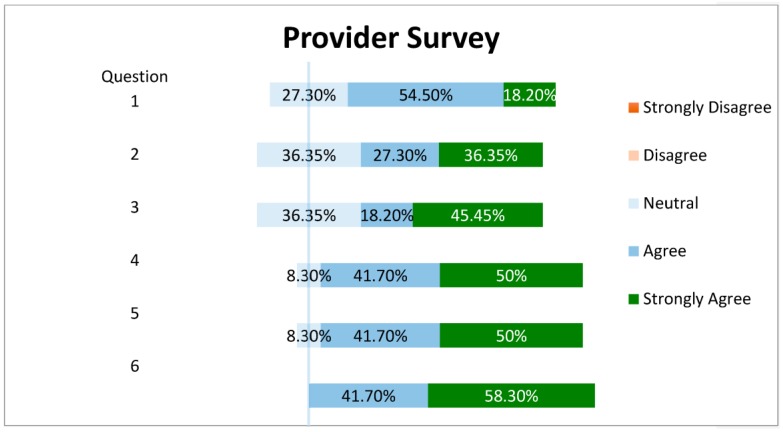
Results of provider survey for pharmacist CMR service.

Finally, a survey was administered to 14 members of the medical staff of the hosting primary care clinic. A response was received from 12 participants, giving a response rate of 86%. The results of the survey are summarized in Table 4 and Figure 2. Additional feedback comments on the survey included, “I loved having access to a pharmacist for questions on difficult patients with polypharmacy”, and “nice interacting with a medication expert”.

## 4. Discussion

This study found a significant difference in number of medication-related problems for geriatric patients after receiving a comprehensive medication review with a pharmacist compared to patients that did not receive a CMR. This research further acknowledges the pharmacist’s role in medication therapy management services and confirms findings from previous studies [1,2,3,8,10,11,12,13,14,15,16,17]. As CMRs can be billed to Medicare, providing these clinical services will pave the way for future ambulatory pharmacy practice. 

The pharmacist was able to provide a variety of interventions, indicating that with the complexity of geriatric patients, this population is an optimal target to improve medication management. The fact that over 80% of patients had missing or inaccurate medication profiles after the completion of their primary care provider appointment shows the gap that remains in the medication reconciliation process, which the pharmacist was able to fulfill when providing CMRs. While the acceptance rate for the START and STOPP Criteria interventions was lower than anticipated, the overall acceptance rate was typical to rates observed at Bronson Battle Creek inpatient settings for smaller intervention types. This lower rate is most likely attributed to the lack of discontinuation of benzodiazepines, a problematic class of medications, which require tapering to prevent withdrawal effects. More education and frequent discussions on the use of benzodiazepines may be warranted for this non-acute, clinical setting. 

There were no statistically significant differences in emergency department visits or hospitalizations between the two groups. There was a trend observed in that more fall-related emergency department visits occurred in the intervention group. It should be noted that two of the three falls were for the same patient and no falls could directly be attributed to high-risk medications. Healthcare utilization remains a point of interest for further research in hopes of identifying whether CMRs have an impact on acute healthcare utilization and associated costs. 

Finally, ambulatory pharmacy services are greatly limited by the lack of billing opportunities and adoption by physicians as a necessity to their practices. Using a provider survey, we were able to identify that the pharmacist was a positive addition to a family medicine office practice to improve medication regimens, drug-information consults, and provide patient-centered, interdisciplinary care. 

Strengths of this study include the direct integration of a pharmacist into a primary care practice and the positive outcomes resulting from the addition of medication therapy management services. However, it is important to also take note of the limitations of this study. A relatively small population size limited our results with only 26 patients in each group, and a sample size calculation was not performed. Next, the follow-up period was short (90 days) due to the scope of this research project. Further evaluations in the future can expand this time period and follow-up with any potential differences in results. Finally, a lack of scheduling appointments for a comprehensive medication review with the pharmacist presented numerous barriers to the study. Many patients were missed, simply by providers or medical assistants forgetting to have the patient meet with the pharmacist at the end of their appointment. This lack of participation from physicians and mid-level providers remained, despite our education for providers on the purpose and scope of the project. 

## 5. Conclusions

In conclusion, a comprehensive medication review performed by a pharmacist was associated with a statistically significant decrease in the number of medication-related problems defined according to the START and STOPP Criteria, compared with those patients that did not receive a CMR. Pharmacist interventions improved medication use in primary care, however physicians did not necessarily adopt even well-established recommendations. Despite these shortfalls, the presence of a pharmacist to assist in medication management was well supported by the primary care practice. Our hope is that this project provides direction and guidance for one method to establish an ambulatory pharmacist CMR service. 

## Figures and Tables

**Table 1:** Demographic Information.

	CMR (*N* = 26)	Control (*N* = 26)	*p*-Value
Age	76.4 ± 7.7	76.5 ± 8.5	0.49
Male (%)	10 (38%)	9 (35%)	0.68
# Medications	14.2 ± 5.4	12.0 ± 4.4	0.061
SCr	0.93 ± 0.38	0.86 ± 0.29	0.22
BZDs	6	6	-

CMR: Comprehensive Medication Review; SCr: serum creatinine; BZDs: benzodiazepines.

**Table 2:** Primary Outcome—Number of Medication-related Problems.

	CMR (*N* = 26)	Control (*N* = 26)	*p*-Value
START	4	3	-
STOPP	7	21	-
Total	11	24	0.002

CMR: Comprehensive Medication Review.

**Table 3:** Acute Healthcare Utilization.

	CMR	Control	*p*-Value
ED Visits (total)	7	6	0.413
Fall-related	3	0	0.100
Medication-related	1	1	-
Hospitalizations (total)	3	4	0.379
Fall-related admissions	0	0	-
Medication Related	1	1	-
Mean Length of Stay (days)	4.33	3	

CMR = comprehensive medication review group.

**Table 4:** Provider Survey.

Question
1. Our providers/I benefited from utilizing the pharmacist within our medical practice
2. Our patients benefited from a comprehensive medication review by the pharmacist
3. The pharmacist was a positive attribute to our medical practice
4. Utilizing an interdisciplinary approach provides for patient-centered healthcare
5. I would consult with a pharmacist (if available) to assess medication therapy for my/our patients
6. Medication management is a difficult task for our elderly patients
